# Bibliometric and Visualization Analysis of Human Coronaviruses: Prospects and Implications for COVID-19 Research

**DOI:** 10.3389/fcimb.2020.581404

**Published:** 2020-09-23

**Authors:** Ziqin Deng, Junsheng Chen, Ting Wang

**Affiliations:** Department of Pathogen Biology, School of Basic Medicine, Tongji Medical College, Huazhong University of Science and Technology, Wuhan, China

**Keywords:** human coronavirus, COVID-19, SARS-CoV-2, bibliometric, visualization

## Abstract

Human coronaviruses, which can cause a range of infectious diseases, have been studied for nearly 60 years. The field has gained renewed interest from researchers around the world due to the COVID-19 outbreak in late 2019. Despite a large amount of research, little is known about the knowledge structure and developing trends of this topic. Here, we apply bibliometric analysis along with visualization tools to analyze 15,207 publications related to human coronavirus from the Scopus database, using indicators on publication and citation, journal, country or territory, affiliation and international cooperation, author, and keyword co-occurrence cluster. The results show that research on human coronavirus is dominated by SARS-CoV. Although there have been many publications, only 626 publications (4.1% of total) have more than 100 citations. The top 20 journals with most publications account for 20.6% of total publications and 41% of total citations. In addition to the United States and some European countries, many Asian and African countries are involved in this research, with China holding an important position in this area. Leading researchers from various fields of human coronavirus research are listed to facilitate collaboration and promote effective disease prevention and control. The keywords co-occurrence analysis reveals that the research focus on virology, public health, drugs and other hotspot fields, and uncovers changes in the direction of coronavirus research. The research map on human coronavirus obtained by our analysis are expected to help researchers to efficiently and effectively explore COVID-19.

## Introduction

Coronaviruses, which were discovered in the 1930s (Estola, 1970) and cause a range of respiratory and intestinal infections in animals and humans (Geller et al., 2012), are enveloped viruses with positive-sense single-strand RNA which belong to the *Nidovirales* order, the *Coronaviridae* family and the *Coronavirinae* subfamily (Gonzalez et al., 2003). The name “coronavirus” is derived from the Latin corona, meaning crown or halo, and refers to the virus's characteristic appearance under an electron microscope, which appears like a crown or solar corona (Almeida and Tyrrell, 1967). There are four genera contained in the *Coronavirinae* subfamily: the *Alpha*-, *Beta*-, *Gamma*-, and *Deltacoronavairus*. These viruses can infect not only birds (gamma- and deltacoronaviruses), but also a range of mammalian species (mainly alpha- and betacoronaviruses), including humans (Corman et al., 2018). Coronaviruses have been known for more than 80 years (Saif, 2004), while only seven of them have been proven to infect humans so far. Plenty of evidence has strongly suggested that human coronaviruses exist with or origin from livestock or some wild animals (Corman et al., 2018). In this case, diseases caused by coronaviruses can also be defined as a kind of zoonosis.

Human coronavirus (HCoV) had not been considered as a highly pathogenic virus to humans until the Severe Acute Respiratory Syndrome (SARS) outbreak in 2002 (Peiris et al., 2003) and the Middle East Respiratory Syndrome (MERS) outbreak in 2012 (Zumla et al., 2015). Recently, another coronavirus disease, the COVID-19, caused by a novel coronavirus, SARS-CoV-2 (Ciotti et al., 2019; Tan et al., 2020), has evoked these painful memories and made people more intensely aware of the pathogenic potential of these microorganisms (Cui et al., 2019). This is the seventh coronavirus identified so far to infect humans, with the others being HCoV-229E, HCoV-OC43, HCoV-NL63, HCoV-HKU1, SARS-CoV, and MERS-CoV (Geller et al., 2012). HCoV-229E, HCoV-OC43, HCoV-NL63, and HCoV-HKU1 are able to cause common cold in humans and majority of these infections only manifest mild symptoms in respiratory system (Mackay et al., 2012; Owusu et al., 2014; Annan et al., 2016). However, SARS-CoV and MERS-CoV are more serious and responsible for high case fatality rates, both of whom belong to genus Beta. Once the case-fatality rate of SARS was ~10% (Cheng et al., 2007), while that of MERS ranged around 35%^1^. Similar to SARS-CoV and MERS-CoV, SARS-CoV-2 is also one of the *Betacoronaviruses*, but with quite unique qualities (Tan et al., 2020; Zhu et al., 2020). The clinical case-fatality rate is not as severe as that of SARS-CoV and MERS-CoV, but it is more infectious than either virus (Chan et al., 2020; Huang et al., 2020). As of 3:05 pm CEST, 2 July 2020, there have been 10,533,779 confirmed cases of COVID-19, including 512,842 deaths, reported to WHO^2^. And the virus had also spread to 213 countries and territories around the world and 2 international conveyances^3^ by August 19, 2020, 09:28 GMT.

Scientists have been studying the human coronavirus since its discovery in the 1960s (Hamre and Procknow, 1966; Bradburne et al., 1967; McIntosh et al., 1967), and more than 15,000 papers related to coronavirus have been published (according to our search) (Supplementary Figure 1). However, researchers need to devote a significant amount of time to reading and identifying relevant work in related fields due to the long research interval, large amount of data, uneven quality of scientific research papers, the presence of unnecessary duplications, and the differences between human coronaviruses and emerging viruses. Therefore, it is particularly urgent to sort out important, effective and meaningful information from large databases in order to guide the scientific research, and promote the proper prevention, control, diagnosis and treatment of human coronavirus.

Bibliometrics, together with novel visualization methods of scientific information, have been reported to be helpful to identify emerging outbreaks of infectious disease. This is particularly true in the current age, where thousands of reports can be easily exchanged between public health specialists and healthcare providers across the internet (Takahashi-Omoe and Omoe, 2012; Unkel et al., 2012). Bibliometrics is also recognized as an essential tool and is widely used in a variety of fields to measure and evaluate scientific research quantitatively and qualitatively (Aggarwal et al., 2016; Romero and Portillo-Salido, 2019; Deng et al., 2020). Therefore, in order to accurately, effectively and systematically reveal connections within the human coronavirus field, our study applied bibliometrics and visualization methods to analyze human coronaviruses-related publications and citations, countries and affiliations, as well as journal performance, author impact and keyword co-occurrence cluster. This study seeks to serve as a valuable reference and guidance for virologists, pharmacists, clinicians and epidemiologists studying the emerging human coronavirus, and to provide novel ideas for finding effective control measures, as well as drugs and vaccines, as soon as possible.

## Methods

### Source Database

Our analysis was conducted using the online database Scopus (https://www.scopus.com/), which provides a comprehensive collection of different types of scientific peer-reviewed literature (Romero and Portillo-Salido, 2019; Bonilla-Aldana et al., 2020). The document search was performed on February 15, 2020.

### Search Strategy

In order to cover as many target documents as possible, we had selected terms that might be used by most scientific publications before the search formula was designed: besides “human coronavirus,” “novel coronavirus” and their abbreviation forms “HCoV” and “nCoV,” we have the terms “human coronavirus 229E” (“HCoV-229E”), “human coronavirus OC43” (“HCoV-OC43”), “human coronavirus NL63” (“HCoV-NL63”), and “human coronavirus HKU1” (“HCoV-HKU1”) to search for publications related to these four currently known non-severely pathogenic human coronavirus; for SARS related publications, we have “severe acute respiratory syndrome-related coronavirus” (“SARSr-CoV” or “SARS-CoV”) and “severe acute respiratory syndrome” (“SARS”); for MERS related publications, we have “Middle East respiratory syndrome-related coronavirus” (“MERS-CoV”) and “Middle East respiratory syndrome” (“MERS”) as well as “MERS coronavirus Erasmus Medical Center/2012” (“MERS coronavirus EMC/2012,” “HCoV-EMC/2012,” or “EMC/2012”), “novel coronavirus-2012” (“2012-nCoV”) and “camel flu”; as for the COVID-19 related publications, we have “severe acute respiratory syndrome coronavirus-2” (“SARS-CoV-2”), “coronavirus disease-2019” (“COVID-19”), “2019-novel coronavirus” (“2019-nCoV”), “2019-nCoV acute respiratory disease,” “novel coronavirus pneumonia” and “novel coronavirus-infected pneumonia.” Several writing formats that are not generally accepted, like “corona virus” and “HCoV229E,” were also taken into consideration after we had found them been used in some publications.

Notably, when directly using the abbreviation “SARS” and “MERS” to search, we were provided with a massive number of unrelated results in other fields. We decided to add qualifiers as limitation to these terms to have the accuracy of search optimized (Table 1).

**Table 1:** Collocation of qualifiers and subjects in the search formula.

**Qualifier^a^**	**Subject**	**Target term covered**
(Not applicable)	“Human coronavirus” OR “Human corona virus” OR HCoV OR HCoV229E OR HCoVOC43 OR HCoVNL63 OR HCoVHKU1	Human coronavirus Human coronavirus 229E (HCoV-229E) Human coronavirus OC43 (HCoV-OC43) Human coronavirus NL63 (HCoV-NL63) Human coronavirus HKU1 (HCoV-HKU1) HCoV-EMC/2012
Coronavirus OR “Corona virus” OR “*CoV”	229E OR OC43 OR NL63 OR HKU1	Coronavirus 229E (CoV-229E) Coronavirus OC43 (CoV-OC43) Coronavirus NL63 (CoV-NL63) Coronavirus HKU1 (CoV-HKU1)
(Not applicable)	“Novel coronavirus” OR “Novel corona virus” OR nCoV	Novel coronavirus Novel coronavirus pneumonia Novel coronavirus-infected pneumonia Novel coronavirus-2012 Novel coronavirus-2019 2012 novel coronavirus 2019 novel coronavirus 2012-nCoV 2019-nCoV 2019-nCoV acute respiratory disease
“*CoV” OR *virus OR Crisis OR Outbreak OR Epidemic OR Pandemic	{SARS} OR {MERS}	SARS SARS-CoV SARS-CoV-2 SARS-like virus MERS MERS-CoV MERS coronavirus Erasmus Medical Center/2012 MERS coronavirus EMC/2012
(Not applicable)	“Severe acute respiratory syndrome” OR “SARSr-CoV”	Severe acute respiratory syndrome Severe acute respiratory syndrome-related coronavirus Severe acute respiratory syndrome coronavirus-2 SARSr-CoV
(Not applicable)	“Middle East respiratory syndrome” OR “Camel flu” OR “EMC/2012”	Middle East respiratory syndrome Middle East respiratory syndrome-related coronavirus Camel flu EMC/2012
(Not applicable)	“Coronavirus disease-2019” OR “COVID-19”	Coronavirus disease-2019 COVID-19

a *Subjects with no qualifier to collocate with are tagged as Not applicable*.

And finally, we conducted the search with the following formula, designed according to the search rules of Scopus (Deng et al., 2020): TITLE-ABS-KEY (“human coronavirus” OR “human corona virus” OR HCoV OR “novel coronavirus” OR “novel corona virus” OR nCoV OR “severe acute respiratory syndrome” OR ({SARS} AND “^*^CoV” OR ^*^virus OR crisis OR outbreak OR epidemic OR pandemic) OR “SARSr-CoV” OR “middle east respiratory syndrome” OR ({MERS} AND “^*^CoV” OR ^*^virus OR crisis OR outbreak OR epidemic OR pandemic) OR “camel flu” OR “EMC/2012” OR “coronavirus disease 2019” OR “COVID-19” OR (coronavirus OR “corona virus” OR “^*^CoV” AND 229E OR OC43 OR NL63 OR HKU1) OR HCoV229E OR HCoVOC43 OR HCoVNL63 OR HCoVHKU1) (Table 1).

### Data Synthesis and Analysis

All search results, data and information that were essential for our analysis, including the count of citations and some indicators, were extracted from Scopus on February 15, 2020. Complete document lists were exported as CSV files, which were then imported into Microsoft Excel 2016 for ranking and counting. Bar charts were made by GraphPad Prism 8, and VOSviewer 1.6.12 was used to generate the visualization maps.

In the analysis of country (or territory), affiliation and author, every co-author was counted equally. In this case, documents with too many authors are not comparable to documents with fewer authors. Since only documents with a maximum of 15 authors were counted by Scopus, we followed the same strategy when conducting the visualization analysis with VOSviewer.

## Results

### Analysis of the Number of Publications and Citations

Our search yielded 15,979 results, based on their titles, abstracts and key words. Then we limited our results to publications in English and Chinese, which yielded 15,207 documents in total, including 9,182 articles, 2,170 reviews and 3,855 documents of other types (Supplementary Figure 1).

**Figure 1:**
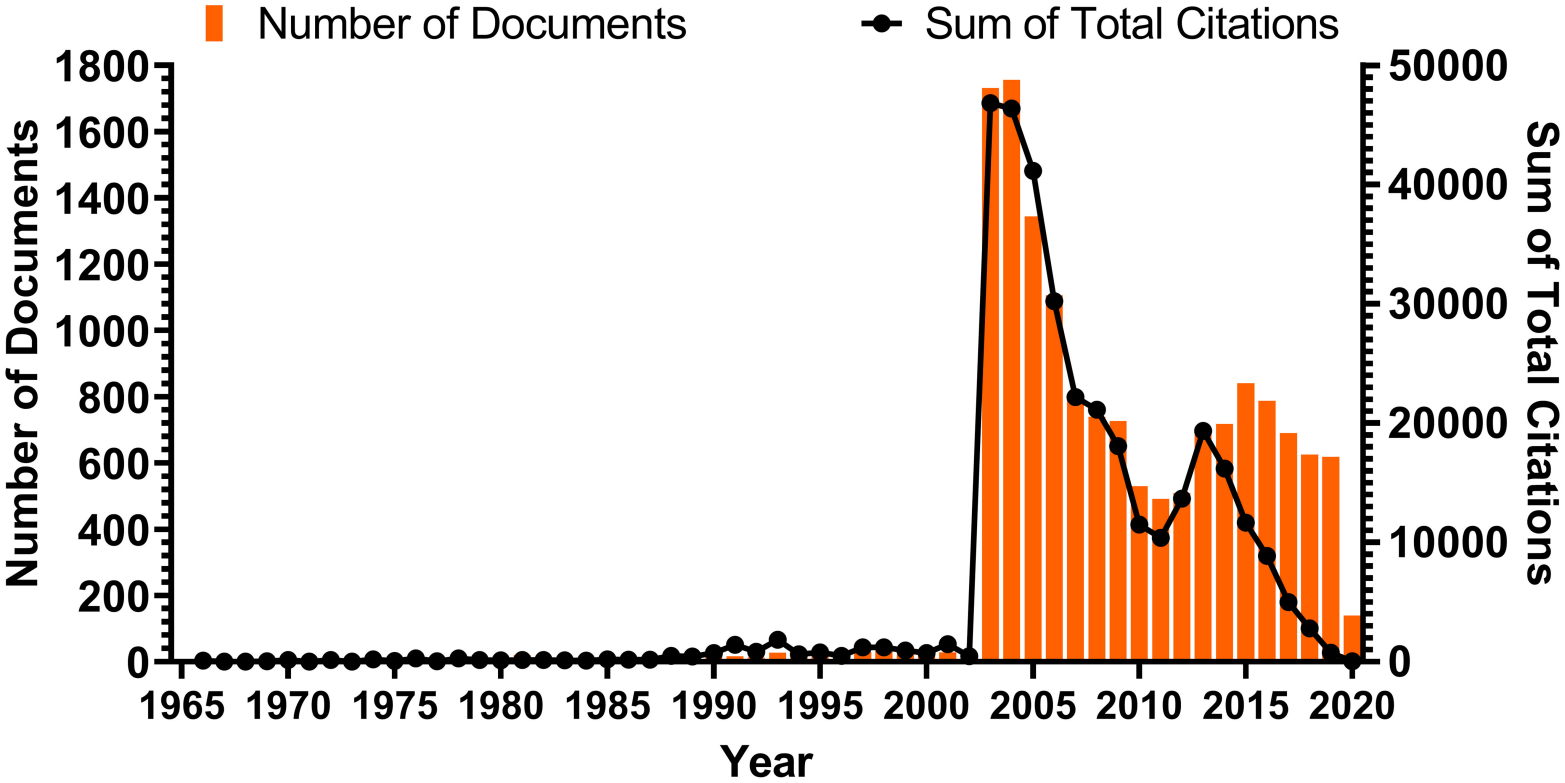
Number of documents published each year and the corresponding citation count of those documents. The bars show the number of documents published each year; the nodes on the line chart represent the sum of total citations that corresponding documents of that year had received. The average number of documents published per year is 276.5.

As expected, the annual publications count was naturally divided into four sections, due to three notable epidemic events in history. Before the outbreak of SARS, the annual publication amount remained low, reaching a maximum of 29 in 1998. It was not until the SARS crisis occurred in 2002 that the scientific publications in this field experienced explosive growth. The annual publication number rose suddenly to 1,729 in 2003 and continued to rise the following year, when it reached 1,754 publications. For the next few years, the publication count declined gradually, reaching a low of 488 publications in 2011. It was the first outbreak of MERS in the Middle East that led to renewed interest in coronavirus and an increase in publications. This lasted for 4 years, during which time the annual number reached another peak, 838 publications in 2015, coinciding with the second outbreak of MERS in South Korea. Notably, the annual publication number has continued to decline since then. For the publications on human coronavirus in 2020, only publications released prior to February 15 were counted. Interestingly, the trend for summed citation of annual publications closely aligned with annual publication production prior to 2013 (Figure 1).

For our citation analysis, we counted the number of publications with different citation amounts. We found that more than half of the publications, up to 9,383 (61.7%, 4,991 articles, 1,075 reviews), had been cited no more than 10 times, including 3,336 that had gone uncited (21.9%, 1,373 articles, 308 reviews). Only 4.1% of publications (626 publications, 432 articles, 161 reviews) had more than 100 citations, and only 206 publications (1.4%) and 46 publications (0.3%) had been cited more than 200 and 500 times, respectively (Supplementary Figure 2).

The top 20 most cited articles and non-articles are listed separately in Table 2 and Supplementary Table 1. Among the top 20 articles, six were published in the New England Journal of Medicine, four in Science, three in Nature, two in the Lancet and the remaining five articles were published in five other journals. In total, these 20 articles were published in nine different journals. Most of them were published after 2002, corresponding to the time of the SARS outbreak. The research areas of the top 20 articles involve public health, preventive medicine, epidemiology, clinical reports and virological study, including the identification, isolation, and analysis of natural hosts of these coronaviruses.

**Table 2:** Top 20 articles with the most citations.

**Rank^a^**	**Title**	**Total citations**	**Authors^b^**	**Source**	**Year**
1	Global trends in emerging infectious diseases	2,686	Jones et al.	Nature	2008
2	A novel coronavirus associated with severe acute respiratory syndrome	2,156	Ksiazek et al.	New England Journal of Medicine	2003
3	Identification of a novel coronavirus in patients with severe acute respiratory syndrome	1,999	Drosten et al.	New England Journal of Medicine	2003
4	Coronavirus as a possible cause of severe acute respiratory syndrome	1,658	Peiris et al.	Lancet	2003
5	Characterization of a novel coronavirus associated with severe acute respiratory syndrome	1,572	Rota et al.	Science	2003
6	The genome sequence of the SARS-associated coronavirus	1359	Marra et al.	Science	2003
7	Isolation of a novel coronavirus from a man with pneumonia in Saudi Arabia	1,339	Zaki et al.	New England Journal of Medicine	2012
8	A major outbreak of severe acute respiratory syndrome in Hong kong	1,217	Lee et al.	New England Journal of Medicine	2003
9	Psychological stress and susceptibility to the common cold	1,103	Cohen et al.	New England Journal of Medicine	1991
10	Angiotensin-converting enzyme 2 is a functional receptor for the SARS coronavirus	1,047	Li et al.	Nature	2003
11	Isolation and characterization of viruses related to the SARS coronavirus from animals in Southern China	1,013	Guan et al.	Science	2003
12	Guidelines for preventing health-care-associated pneumonia, 2003: recommendations of CDC and the Healthcare Infection Control Practices Advisory Committee	978	Tablan et al.	MMWR. Recommendations and reports: Morbidity and mortality weekly report. Recommendations and reports/Centers for Disease Control	2004
13	Clinical progression and viral load in a community outbreak of coronavirus-associated SARS pneumonia: A prospective study	962	Peiris et al.	Lancet	2003
14	Interfering with disease: A progress report on siRNA-based therapeutics	941	de Fougerolles, A et al.	Nature Reviews Drug Discovery	2007
15	2007 Guideline for Isolation Precautions: Preventing Transmission of Infectious Agents in Health Care Settings	934	Siegel et al.	American Journal of Infection Control	2007
16	Bats are natural reservoirs of SARS-like coronaviruses	918	Li et al.	Science	2005
17	Superspreading and the effect of individual variation on disease emergence	896	Lloyd-Smith et al.	Nature	2005
18	Identification of severe acute respiratory syndrome in Canada	861	Poutanen et al.	New England Journal of Medicine	2003
19	Respiratory viruses and exacerbations of asthma in adults	855	Nicholson et al.	British Medical Journal	1993
20	Identification of a new human coronavirus	818	Van Der Hoek et al.	Nature Medicine	2004

a *Ranked by total citations*.

b *The first authors showed up (on Scopus) were provided. This doesn't mean this author contributed the most*.

### Analysis of Journal

All English and Chinese documents came from 3,443 different journals. The 21 journals with the most publications on human coronavirus are listed in Table 3. This list includes four journals that published articles that were in the top 20 article list (Table 2) (New England Journal of Medicine, Science, Nature and Lancet). These 21 journals produced 20.6% (3,126) of all publications analyzed here and 41.0% of the total citations (140,450 of 342,451). Among them, the New England Journal of Medicine had the highest values in two indicators, citations per document and CiteScore (2018). Also, notably, contributions made by Chinese journals like Hong kong Medical Journal and Chinese Medical Journal cannot be ignored. Figuring out core journals in a specific research field is of considerable importance. This can not only help academic achievements be known and used as much as possible, but also provide those who need the information with more concentrated access.

**Table 3:** Top 20 journals with the most documents.

**Rank^a^**	**Journal**	**Document numbers**	**Total citations^b^**	**Citations per document^c^**	**CiteScore 2018^d^**
1	Journal of Virology	482	24,768	51.4	4.02
2	Emerging Infectious Diseases	340	12,634	37.2	4.46
3	Lancet	221	13,513	61.1	10.28
4	Plos One	205	4,199	20.5	2.97
5	Virology	156	4,952	31.7	3.29
6	Science	140	11,058	79.0	15.21
7	Nature	134	9,885	73.8	15.21
8	Journal of Infectious Diseases	128	4,468	34.9	4.10
9	Clinical Infectious Diseases	122	5,083	41.7	5.31
10	Lancet Infectious Diseases	120	4,247	35.4	6.53
11	Advances In Experimental Medicine And Biology	109	711	6.5	1.71
12	Journal of General Virology	108	3,781	35.0	2.78
13	Journal of Medical Virology	107	3,380	31.6	1.94
14	Viruses	104	1,244	12.0	4.03
15	Proceedings of The National Academy of Sciences of The United States of America	100	10,387	103.9	8.58
16	Antiviral Research	99	2,257	22.8	4.19
17	New England Journal of Medicine	93	15,609	167.8	16.10
18	Vaccine	92	2,113	23.0	3.18
19	Hong kong Medical Journal	92	397	4.3	0.70
20	Journal of Clinical Microbiology	88	5,262	59.8	3.65
21^e^	Chinese Medical Journal	86	502	5.8	1.16

a *Ranked by document number*.

b *Total citations mean the sum of citations received these years (before our research time) about those documents (the Document' numbers column in this table) in each journal*.

c *Citations per document was calculated according to the documents' numbers and their total citations*.

d *The CiteScore in 2019 or 2020 has not been provided on Scopus when this research was conducted on February 15, 2020*.

e *“Chinese Medical Journal” ranked 21, because “Vaccine” (ranked 18) and “Hong kong Medical Journal” (ranked 19) has the same document number (then ranked by total citations)*.

### Analysis of Country (or Territory), Affiliation, and International Cooperation

**Figure 2:**
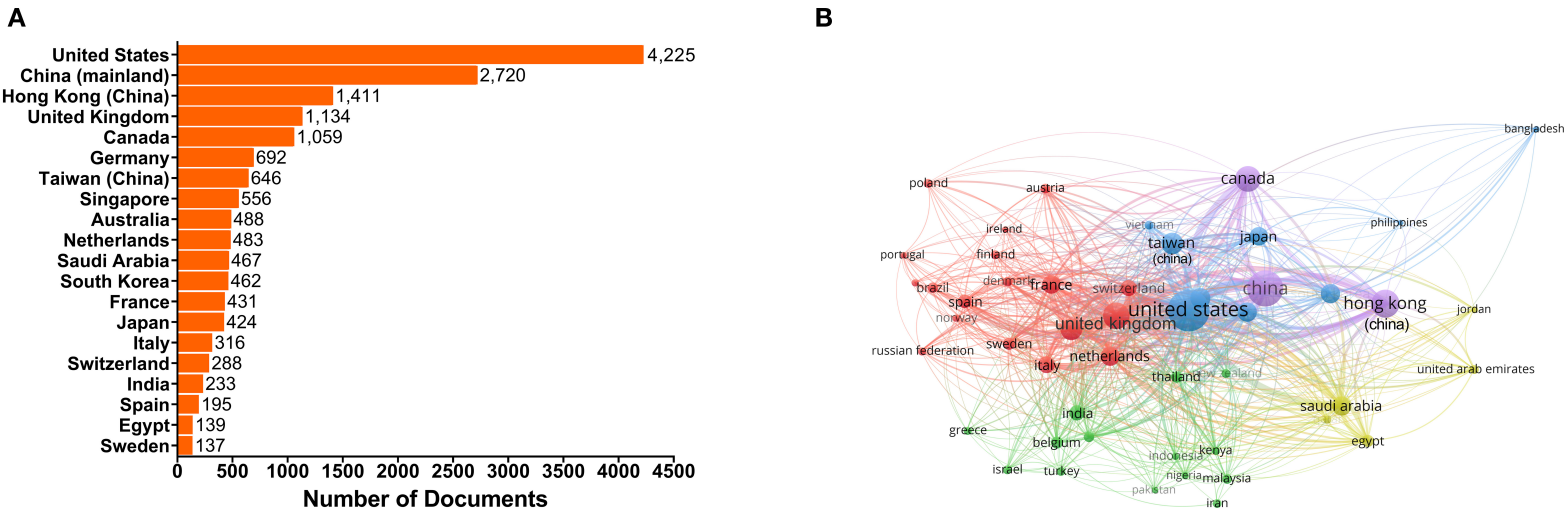
Top 20 countries or territories with the most documents and country or territory co-authorship analysis map. **(A)** Top 20 countries or territories with the most publications. The bars show the number of documents produced by the country or territory. **(B)** Country or territory co-authorship analysis map. The size of each node indicates the number of documents produced by the country or territory. The thickness of each link indicates the strength of collaboration relationship between two countries or territories. The distance between two nodes indicates the relatedness of the links they each has. Nodes with some common attributes are assigned to a cluster and are color-coded.

The United States has dominated this field with 143,960 citations on its 4,225 published documents. China ranked second with a total of 49,316 citations for its 2,720 documents from researchers in mainland China, 48,828 total citations on 1,411 documents from researchers in China Hong kong and 13,408 total citations on 646 documents from researchers in China Taiwan. The United Kingdom and Canada have also contributed significantly to this research area (Figure 2A, Supplementary Table 2). The United States, China and the United Kingdom are the three most active countries, and all have a high frequency of international cooperation, according to the international cooperation network (Figure 2B).

**Figure 3:**
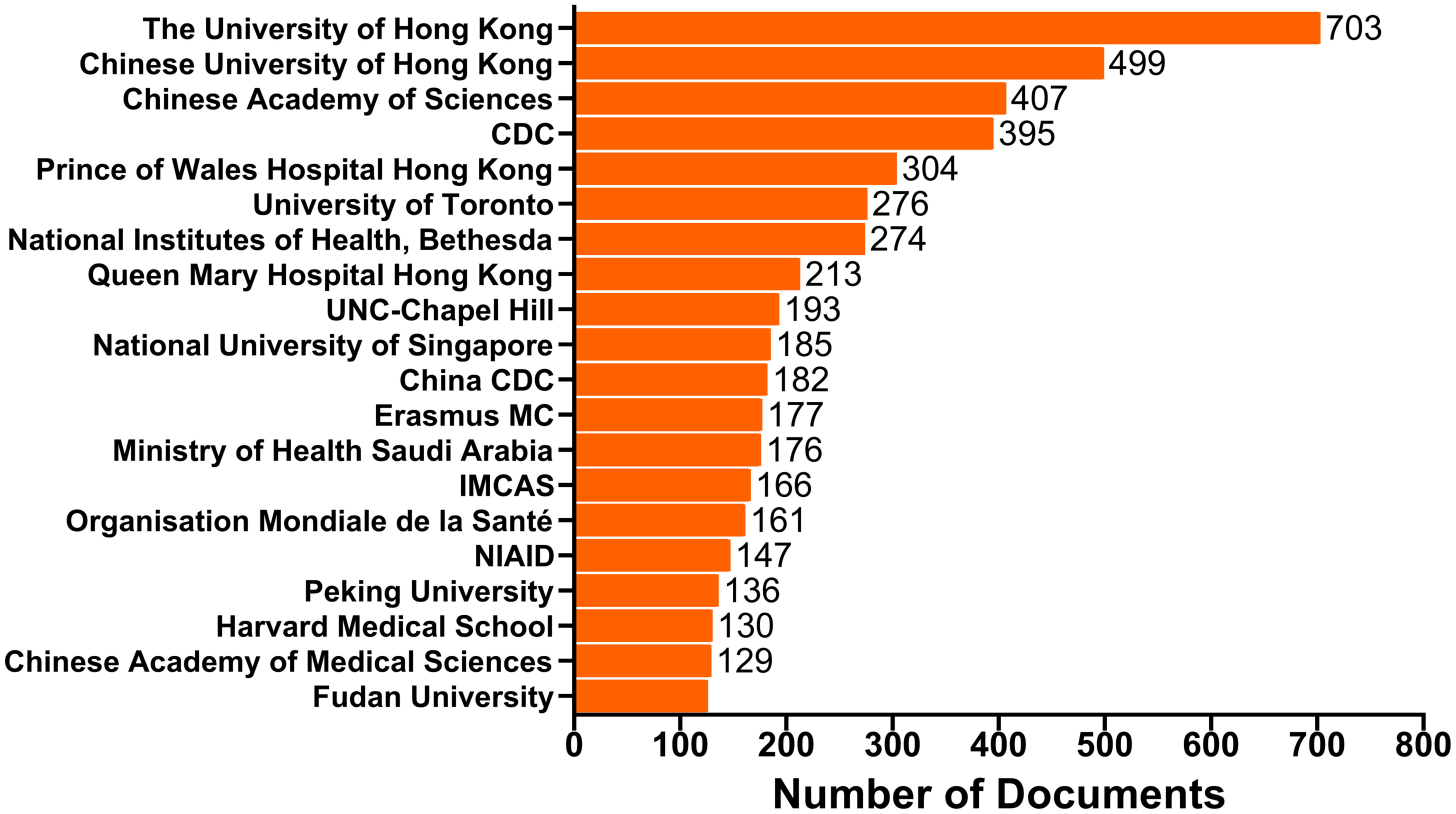
Top 20 affiliations with the most publications. The bars show the number of documents produced by the affiliation. CDC, Centers for Disease Control and Prevention; UNC-Chapel Hill, The University of North Carolina at Chapel Hill; China CDC, Chinese Center for Disease Control and Prevention; IMCAS, Institute of Microbiology Chinese Academy of Sciences; NIAID, National Institute of Allergy and Infectious Diseases.

It is worth noting that China has the top position in terms of our affiliation analysis, with the University of Hong kong publishing 703 documents, Chinese University of Hong kong contributing 499 documents, Chinese Academy of Sciences producing 407 documents, Prince of Wales Hospital Hong kong publishing 304 documents, etc. (Figure 3, Supplementary Table 3).

### Analysis of Author

In Table 4, we listed the top 20 authors with the most documents. Yuen Kwok-yung at The University of Hong kong, Shenzhen Hospital, was the most productive researcher, publishing 180 documents associated with human coronavirus. Christian Drosten at Charité-Universitätsmedizin Berlin ranked second, with 128 documents. Joseph S.M. Peiris at The University of Hong kong ranked the third, with 111 documents and an h-index of 108. Though they did not produce as many documents, Joseph Sung Jao-yiu, with an h-index 120, and Albert D.M.E. Osterhaus, with an h-index 113, also have prominent places in this field. Additionally, the contributions from Kwok-Hung Chan, Leo L.M. Poon and Guan Yi, which have all garnered high citation counts, cannot be ignored. The studies of those active and emerging researchers with unique ideas rather deserves our attention. It inspired us that more comprehensive and detailed evaluation of researchers will help to identify their specific areas of expertise, and therefore make their work more valued when applicable.

**Table 4:** Top 20 authors with the most documents.

**Rank^a^**	**Author**	**Documents' numbers**	**Total citations^b^**	**Citations per document^c^**	**Affiliation^d^**	**Author's h-index**	**Documents' h-index^e^**
1	Yuen, Kwok Yung	180	15,591	86.6	The University of Hong kong, Shenzhen Hospital, Shenzhen, China	97	62
2	Drosten, Christian	128	11,438	89.4	Charité – Universitätsmedizin Berlin, Berlin, Germany	71	47
3	Peiris, Joseph S.M.	111	12,317	111.0	The University of Hong kong, Pokfulam, Hong kong (China)	108	47
4	Al Memish, Ziad	108	5,908	54.7	Ministry of Health Saudi Arabia, Riyadh, Saudi Arabia	77	41
5	Baric, Ralph S.	95	4,965	52.3	The University of North Carolina at Chapel Hill, Chapel Hill, United States	73	40
6	Jiang, Shibo	94	3,223	34.3	Fudan University, Shanghai, China	58	33
7	Chan, Paul KS	91	3,950	43.4	Chinese University of Hong kong, Shatin, Hong kong (China)	63	32
8	Woo, Patrick C.Y.	90	5,877	65.3	The University of Hong kong, Pokfulam, Hong kong (China)	64	37
9	Sung, Joseph Jao Yiu	88	4,278	48.6	Chinese University of Hong kong, Shatin, Hong kong (China)	120	33
10	Perlman, Stanley	87	3,367	38.7	Children's Hospital of Iowa, Iowa City, United States	48	33
11	Al-Tawfiq, Jaffar Ali	78	3,020	38.7	Johns Hopkins Aramco Healthcare, Dhahran, Saudi Arabia	38	28
12	Haagmans, Bart L.	75	4,750	63.3	Erasmus MC, Rotterdam, Netherlands	52	34
13	Chan, Kwok Hung	74	8,127	109.8	The University of Hong kong, Pokfulam, Hong kong (China)	67	44
14	Poon, Leo L.M.	73	9,613	131.7	The University of Hong kong, Pokfulam, Hong kong (China)	73	43
15	Lau, Susanna K.P.	69	5,153	74.7	The University of Hong kong Li Ka Shing Faculty of Medicine, Hong kong (China)	58	35
16	Guan, Yi	67	10,375	154.9	State Key Laboratory of Emerging Infectious Diseases, China	96	46
17	Du, Lanying	66	2,092	31.7	New York Blood Center, New York, United States	33	30
18	Talbot, Pierre J.	65	1,689	26.0	INRS-Institut Armand Frappier, Laval, Canada	30	25
19	Müller, Marcel Alexander	63	4,398	69.8	German Centre for Infection Research (DZIF), Berlin, Germany	43	33
20	Osterhaus, Albert D.M.E.	61	9,112	149.4	Artemis One Health, Utrecht, Netherlands	113	33

a *Ranked by document number*.

b *Total citations mean the sum of citations received these years (before our research time) about those documents (the Documents' numbers column in this table) of each author*.

c *Citations per document was calculated according to the documents' numbers and their total citations*.

d *Affiliation of each author is the latest one shown on Scopus*.

e *Documents' h-index extracted from Scopus is the h-index of those documents (the Documents' numbers column in this table) written by the author. It means there are “h” documents have been cited at least “h” times*.

### Analysis of Keyword Co-occurrence Cluster

**Figure 4:**
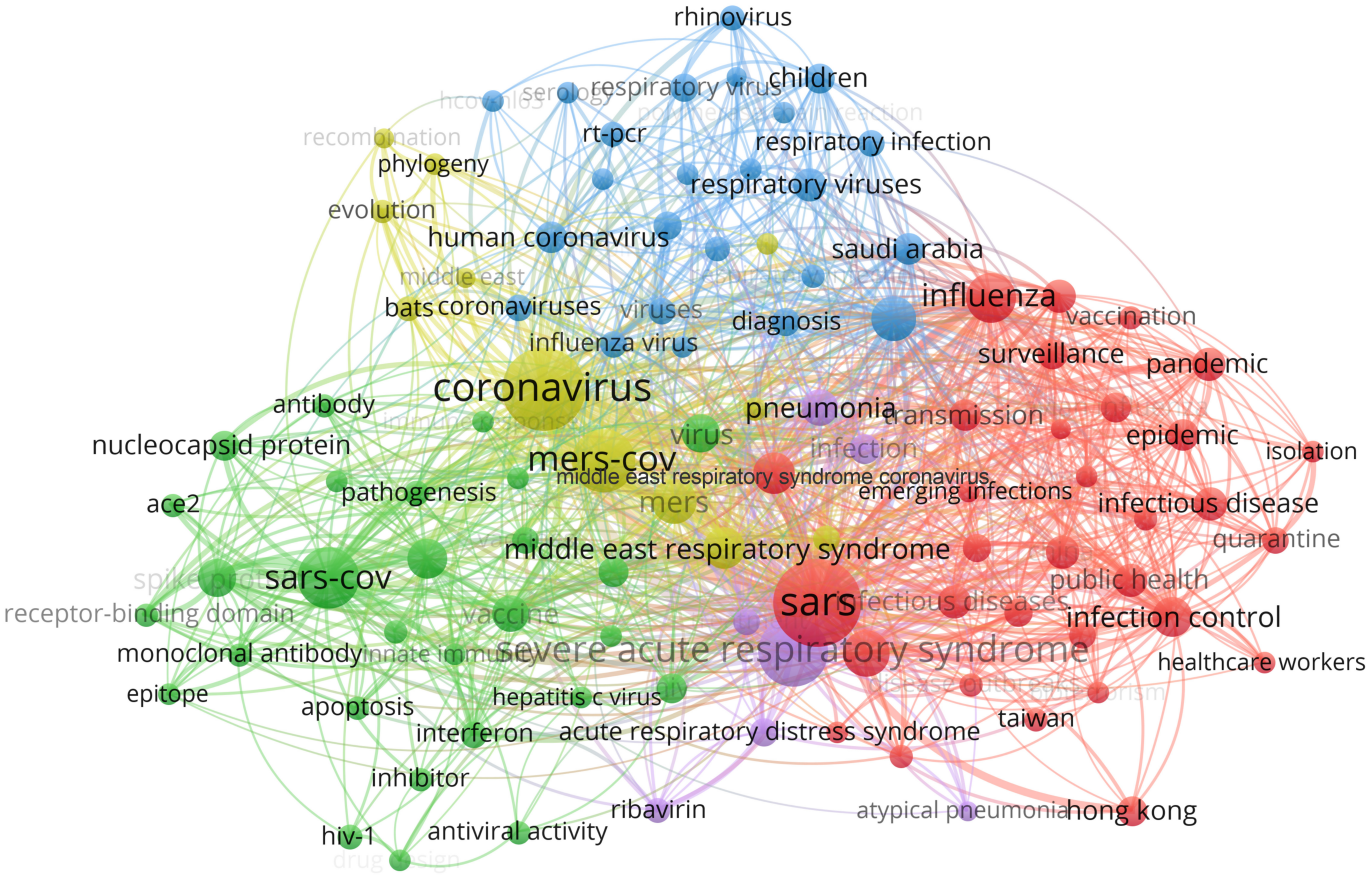
Keyword co-occurrence analysis map. The size of each node indicates the occurrence of the keyword in all 15,207 documents. The thickness of each link indicates the strength of co-occurrence relationship between two keywords. The distance between two nodes indicates the relatedness of the links they each has. Nodes with common attributes are assigned to a color-coded cluster.

A co-occurrence relationship is formed between two keywords when they both occurred in the same paper. Keywords with strong co-occurrence relationship can reveal research hotspots more precisely than a single keyword. In our visual representation, strongly connected keywords were colored the same, indicating that they might share something in common. Keywords shown in red are roughly connected to public health, preventive medicine and epidemiology. According to these keywords, human coronavirus diseases like “SARS,” “MERS” and COVID-19 may have something worthwhile for comparison with other “infectious diseases” like “influenza” in their epidemiological characteristics; “healthcare workers,” “transmission,” “surveillance,” “quarantine,” or “isolation” may be the focuses of these studies, which can help to promote current disease control and prevention measures. Keywords in blue and yellow are related to virus detection and clinical diagnosis. Among them, “serology” and “RT-PCR” are likely to be the methods, the research objects are mainly some kinds of “respiratory viruses,” and the research purposes are mostly on “evolution,” “phylogeny,” and “diagnosis.” The words in green are mainly virology-related and also include some immunological and pharmaceutical research: “spike protein,” “nucleocapsid protein,” “receptor-binding domain,” “ACE-2” and “apoptosis” are mainly on “pathogenesis,” while “epitope,” “antibody,” “vaccine,” “inhibitor,” “interferon,” “ribavirin,” and “antiviral activity” are mainly about antiviral solutions (Figure 4). With keyword co-occurrence analysis, we can not only figure out the hotspots, but also discover the limitations, and even come up with new ideas.

## Discussion

With the outbreaks of SARS in 2002 and MERS in 2012, along with current COVID-19 pandemic (Harapan et al., 2020), the world has battled three serious human coronavirus outbreaks during the twenty first century. In particular, the COVID-19 outbreak has surpassed the previous two ones in terms of its transmission scale, largely due to the strong infectivity and long asymptomatic incubation period of the emerging pathogen (Epidemiology Working Group for NCIP Epidemic Response, Chinese Center for Disease Control and Prevention, 2020). Human coronavirus has been an area of concern for many years, with the first study conducted almost 60 years ago and plenty of scientific documents published in the decades since. However, when the novel virus emerged, controlling the spread of this virus effectively and promptly remained a significant challenge. This is partially due to the unclear knowledge map of human coronavirus research and an inadequate understanding of the present research status, hotspots and development trends. This can result in a large amount of repetitive, insignificant or inefficient research work, which can prevent effective analysis of this virus and further delay the development of targeted prevention and control measures. Therefore, we applied an innovative bibliometric and visualization analysis to sort and analyze more than 15,000 human coronavirus-related scientific publications spanning 60 years, with the purpose of mapping and managing previous research in this field.

The trends in the annual number of publications and summed citation of annual publications on human coronaviruses (Figure 1) reflect the interest and development of this area over the years (Durieux and Gevenois, 2010). From 1965 to 2002, the number of publications was low and remained at a stable amount. Strikingly, two explosive growth peaks appear separately in 2003 and 2012, matching the onset of the SARS and MERS outbreaks. This sharp growth seen at these times is reflective of the severe impact of emerging coronaviruses on human health. The number of publications is directly proportional to the extent and spread of the infective disease outbreak (Hurtado et al., 2018). The peak that emerged during the SARS crisis is significantly higher than the one that emerged during the MERS outbreak, which is due to the more widespread outbreak and more extensive impact of SARS. Considering the severity of the COVID-19 outbreak, we can predict that this trend will continue and a large amount of publications will follow. Meanwhile, after the short MERS outbreak, the summed citation of annual publications dropped quickly and became out of sync with the number of annual publications, which might reflect a gradual declining interest in human coronavirus research. However, it is believed that the COVID-19 outbreak caused by the emerging SARS-CoV-2 will bring the research on human coronavirus back to forefront. Just as we predicted, the number of literatures on SARS-CoV-2 and COVID-19 has doubled in the past 6 months, according to our latest online-searching in August. This has proven that the consequences of COVID-19 are so catastrophic and far-reaching that it has attracted attention worldwide.

The citation number of an article represents the extent of its dissemination and influence, and thus partly reflects its quality as well (Muniz et al., 2018). Although there have been over 15,000 publications on human coronavirus, only 626 (4.1%) have more than 100 citations, while 9,383 (61.7%) have <10 citations (Supplementary Figure 2). This indicates that although there has been much research on human coronavirus, there might be only a small percentage of these studies that are of high quality. It also indicates that the research field is wide but not deep, implying that much remains for researchers to study about this virus, and in-depth exploration in some specialized areas could shift the focus and direction of this field for the future. Among the top 20 articles listed in Table 2, six were published in the New England Journal of Medicine, four in Science, three in Nature, two in the Lancet and the other five articles were published in five different journals. These nine different journals are very responsive to novel emerging viruses and related diseases. Moreover, 18 of these top articles are related to the SARS outbreak and only one is connected to the MERS outbreak. For the top 20 non-article publications listed in Supplementary Table 1, there were up to 18 different journals in the list, indicating the attention received by these publications, all of which are related to SARS research, from other sources. Therefore, SARS-CoV plays an important and enlightening role in analyzing the research on human coronavirus.

Despite the fact that all English and Chinese publications were obtained from 3,443 different sources, the top 20 journals with the most published documents account for 20.6% of all publications and 41.0% of all citations. This is reflective of the authority of these journals, as well as their high degree of interest in research related to human coronaviruses. Due to the limitations of Scopus, journals can only be sorted by the total number of published documents. However, as shown in Table 3, the New England Journal of Medicine, Proceedings of the National Academy of Sciences of the United States of America, Science, Nature and the Lancet rank in the top five based on citations per document, which is consistent with the results in Table 2. Popular journals and their research trends in a particular field provide researchers with reliable references. In addition, core journals provide researchers with faster search routes and can serve as an important publication guide (Zhuang et al., 2014; Wang et al., 2019).

The United States, the United Kingdom, Germany and other European countries are usually the most active countries at the forefront of scientific research (Sweileh, 2017). However, as can be seen from Figure 2A, the situation is quite different in the human coronavirus field. The United States remains dominant in this field, while some Asian and African countries, such as China, Singapore, Saudi Arabia, South Korea, Japan, India and Egypt ranked in the top 20 for human coronavirus-related research. This implies that the research on emerging viruses is no longer limited within those developed countries with more developing countries getting involved. One possible factor lies in the wide and fast spread of the diseases, which has enabled more and more people have come to realize the urgency of taking timely actions and having a clear understanding of emerging infectious diseases. Additionally, advancements in science and technology, as well as increased funding support from national policies in these regions have been instrumental in discovering and analyzing emerging viral pathogens. In particular, the top 20 affiliations with the highest number of documents published (Figure 3, Supplementary Table 3) reflect Chinese scientists' contributions to the human coronavirus field, with nearly a half of the top 20 affiliations coming from China. Similarly, a recent article from Stanley Perlman also praised the contribution of Chinese scientists to the study of novel human coronaviruses (Perlman, 2020). Here, we generated a map to show the contributions of different countries to the human coronavirus field and display the cooperative relationship between countries (or territories) intuitively (Figure 2B). This map indicates that international cooperation efforts between coronavirus researchers are led by the United States, China, China Hong kong, the United Kingdom and Canada. These results can provide significant guidance for initiating collaborative projects, project applications, and academic exchanges, as well as providing information that can be used in the political sphere in different countries, territories and affiliations concerned with the impact of human coronavirus (Fiala, 2012). As for the COVID-19 pandemic, the lack of information sharing and collaborative efforts is very detrimental to the prevention of diseases. Countries and affiliations are supposed to take on the responsibilities to strengthen mutual trust and international cooperation, so that we can fight against this fatal virus with more joint efforts.

Analyses of author can help to understand a research field in a more comprehensive manner, and to evaluate the contributions of researchers, as well as their research level and academic status in this field, objectively (Podsakoff et al., 2008). In our study, the top 20 authors with the most publications were used to screen out those active researchers in this area. As shown in Table 4, Yuen Kwok-yung has produced the largest number of documents, Guan Yi has the highest number of citations per document, and Joseph Sung Jao-yiu currently has the highest h-index. These data and indicators provide different perspectives on the research level and academic authority of each researcher. Interestingly, consistent with Figure 3, Table 4 reveals half of the top 20 researchers are from China, which also reflects the more active state of Chinese researchers on human coronaviruses. It should be noted that among all the authors, some are researchers from research institutions at universities, some are doctors from hospitals, and some are experts from the Centers for Disease Control and Prevention (CDC), etc. With the current COVID-19 outbreak, it is vital for scientists, clinicians and CDC experts to share and exchange information, and to develop a united approach in emerging viral disease outbreak responses and control (Wang et al., 2020a). In addition, we have learned from the COVID-19 outbreak that at the beginning of a sudden outbreak, the allocation of emergency expert personnel and the expertise, authority and experience of these experts both have a significant impact on the spread of the epidemic. Therefore, it is necessary to consult and analyze the literature in order to identify these leaders ahead of another novel human coronavirus emergency. Our study is able to supplement and complement these efforts.

A large amount of meaningful information can be obtained from keyword co-occurrence analysis, which could enable the identification of hotspots and trends, and guide researchers to related topics in their field (Romero and Portillo-Salido, 2019). In Figure 4, four clear research fields within human coronavirus can be seen, which mainly involve aspects of public health, preventive medicine and epidemiology, clinical work and pharmaceutical research. By contrast, the studies on tracing, evolution and animal carriers of human coronavirus account for only a small proportion of the studies performed, suggesting that the research in these areas is still insufficient and there is room for growth. In our study, it is demonstrated that current research on SARS-Cov-2 and COVID-19 are mainly focused on prevention and treatment. Although there is former experience of SARS and MERS to learn from, people are still likely to get overwhelmed at first in face of global public health emergencies like this, for its rapid spread on a grand scale (Peeri et al., 2020). Promoting the correct use of protective masks in public places, encouraging a safe social distance and avoiding crowd gathering are all effective alternatives to prevent large-scale public transmission (Gasmi et al., 2020). And enhancing medical stuff with reasonable allocation of medical resources (Emanuel et al., 2020), separating different diagnosis and treatment areas, and providing centralized isolation areas for mild patients if necessary (Hellewell et al., 2020) can all help to ease the shortage of medical resources and avoid nosocomial infections. With present absence of specific antiviral drugs or vaccines to COVID-19, one vital step for now is to prevent the spread of SARS-Cov-2. Based on reported data, the effective reproductive number (R_o_) of the SARS-CoV-2 was estimated to be 2.2 approximately (Li et al., 2020), which means that each infected individual can transmit the infection to more than two healthy individuals. It has been indicated that SARS-CoV-2 may attach the angiotensin converting enzyme 2 (ACE2) receptor with its spike protein, in the way similar to SARS-CoV, to enter target cells (Lu et al., 2020; Walls et al., 2020). However, it is still not sufficient enough to explain the high infective efficiency. Further research has revealed some possible reasons. On one hand, the case fatality rate of COVID-19 is much lower than that of SARS (Li et al., 2020); on the other hand, X-ray crystal diffraction has implied that the combination between S protein of SARS-CoV-2 and ACE2 is a little stronger than that of SARS-CoV (Lan et al., 2020; Shang et al., 2020). In fact, different from SARS-CoV, significant mutation in the S protein of SARS-CoV-2 has been identified by genomics analysis, and a specific furin-like protease also plays an essential role in recognition, entry, stability and transmission of the coronavirus (Coutard et al., 2020; Drak Alsibai, 2020). In addition, neutralizing antibody against the virus has also been a hotspot in human coronavirus research nowadays. As for to SARS-CoV and MERS-CoV, several types of representative neutralizing antibodies such as monoclonal antibodies, their functional antigen-binding fragment and the single-chain variable region etc., has been found to block the binding between receptor-binding domain(RBD) region of S protein with ACE2 receptor and then to inhibit the infection (Jiang et al., 2020). This inspires us that the RBD region may be noteworthy when studying the neutralizing antibodies induced by viruses or vaccines. A recent study has reported a human monoclonal antibody 47D11 which targets a conserved epitope of the SARS-CoV-2 S-S1B domain, and might play a role in the prevention and treatment (Wang et al., 2020b). Convalescent sera have been applied to treating COVID-19, but attention needs to be paid to the phenomenon of antibody-dependent enhancement (ADE) caused by non-neutralizing antibodies which target in non-RBD regions (Du et al., 2009). Although there are a range of studies on the origin and transmission of human coronaviruses, especially SARS-CoV, MERS-CoV, and SARS-CoV-2, current evidences are still not so convincing enough. Despite of this, it is widely accepted so far that these three coronaviruses are able to transmit from person to person. They seem to origin from bats and need some intermediate host to facilitate replication, evolution, and variation, and thus can infect human directly. Civet cats and dromedary camels are supposed to act as intermediate hosts for SARS-CoV and MERS-CoV, respectively (Guan et al., 2003; Azhar et al., 2014). According to genome sequencing and evolutionary analysis, bats are also speculated to be the natural host of SARS-CoV-2 (Guo et al., 2020), while turtles, pangolin and snakes are alternatively possible to serve as the intermediate host for its transmission from animal to human (Liu et al., 2020). Study on the origin of the human coronavirus is extremely significant for better understanding, prevention and control of relevant diseases. As it may become the focus of research on emerging human coronaviruses in the near future, more experts, material and financial resources may be urgently needed. In general, previous research on SARS-CoV and MERS-CoV can provide important guidance for the study of SARS-CoV-2 and speed up the development in therapeutic drugs and vaccines.

Although the keyword co-occurrence analysis shows the hotspots and trends within human coronavirus research, it also indicates that some fields remain unexplored or underexplored. For example, the relationship between human coronavirus and immune metabolism, the application of RNA-seq and single cell sequencing technology in coronavirus research, and the possibility of cocktail therapy in viral treatment have not been studied extensively. Due to the short duration of the recent COVID-19 outbreak, studies on SARS-CoV-2 were insufficient in various fields. Keyword co-occurrence analysis could help researchers studying SARS-CoV-2 by providing abundant potential directions in similar fields with other human coronaviruses and revealing the cross-disciplinary exploration potential.

Today we are experiencing an information data explosion. Because researchers have been studying coronavirus for 60 years, there is a massive and complicated array of data. Thus, being able to benefit from such an unprecedented amount of data without being overwhelmed poses a significant obstacle for researchers, especially when facing the emergency of a novel infectious disease outbreak, such as COVID-19. A bibliometric analysis combined with data visualization is critical for exploring and communicating information effectively, and helping researchers to continue to progress (Wong, 2012). For this reason, we applied bibliometric and visualization methods to analyze 15,207 documents of human coronavirus-related studies from the Scopus database using various indicators. We hope that our study will indicate potential directions for scientists to explore, promote cooperation with other human coronavirus researchers across disciplines, guide emerging researchers toward specialities that have yet to be fully developed, enable the development and use of new technologies in this field, and provide valuable ideas for the prevention, diagnosis and treatment of COVID-19.

## Data Availability Statement

Datasets generated for this study will be made available by the authors, to any qualified researcher upon request.

## Author Contributions

TW designed the study. ZD and JC performed the search. TW, ZD, and JC analyzed the data and wrote the manuscript. All authors contributed to the article and approved the submitted version.

## Conflict of Interest

The authors declare that the research was conducted in the absence of any commercial or financial relationships that could be construed as a potential conflict of interest.

## Footnotes

^1^World Health Organization. *MERS-CoV* (2020). Available online at: https://www.who.int/health-topics/middle-east-respiratory-syndrome-coronavirus-mers/ (accessed August 19, 2020).

^2^World Health Organization. *COVID-19* (2020). Available online at: https://covid19.who.int/ (accessed July 2, 2020).

^3^Worldometer. *Coronavirus* (2020). Available online at: https://www.worldometers.info/coronavirus/ (accessed August 19, 2020).
