# Supplementary material for: Bibliometric and Visualization Analysis of Human Coronaviruses: Prospects and Implications for COVID-19 Research
Source: Front Cell Infect Microbiol. 2020 Sep 23;10:581404. doi: 10.3389/fcimb.2020.581404 (PMC7538618; doi:10.3389/fcimb.2020.581404)
Supplement: Supplementary file 1 [file Data_Sheet_1.docx]

***Supplementary Material***


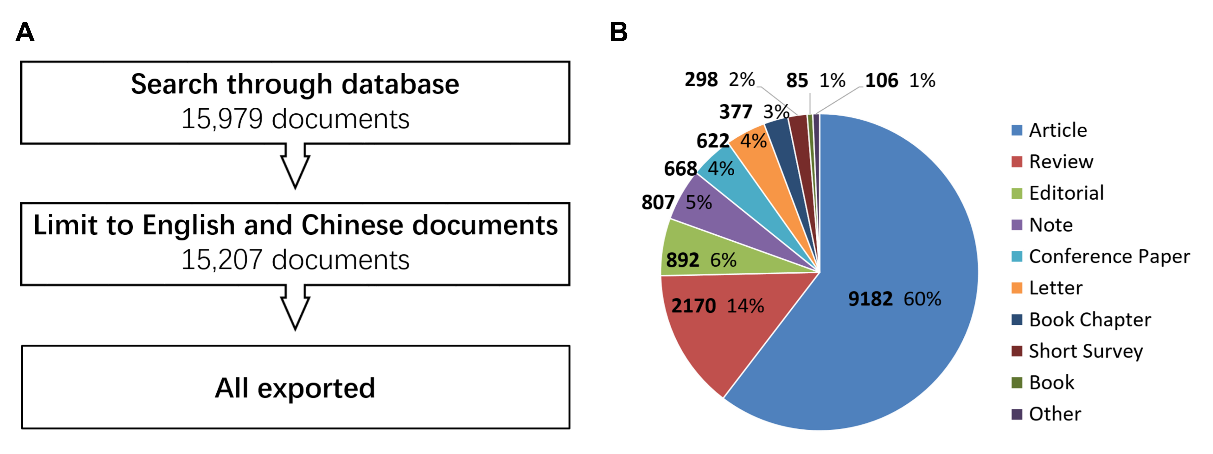


**Supplementary Figure 1.** Data collection process and results. (**A**) Details about the processing of data. (**B**) Document types of all 15,207 English and Chinese results. The numbers represent the count of each type of document and its proportion in all types.


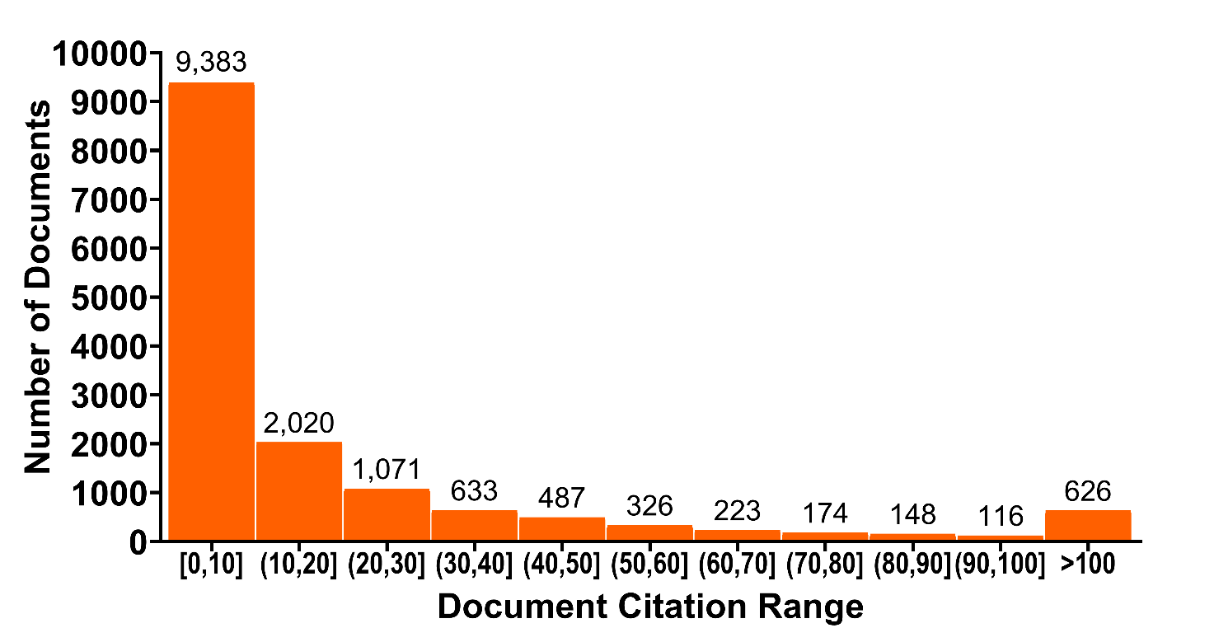


**Supplementary Figure 2.** Distribution of documents’ citation times. The horizontal axis represents the range of citation times. The bars show the number of documents in each interval. The average number of citations per document is 22.5, and the median number is six.

**Supplementary Table 1.** Top 20 non-article type documents with the most citations.

| **Rank^a^** | **Title** | **Document type** | **Total citations** | **Authors^b^** | **Source** | **Year** |
| --- | --- | --- | --- | --- | --- | --- |
| 1 | Knocking down barriers: Advances in siRNA delivery | Review | 1932 | Whitehead, K.A.,  et al. | Nature Reviews Drug Discovery | 2009 |
| 2 | How long do nosocomial pathogens persist on inanimate surfaces? A systematic review | Review | 1119 | Kramer, A.,  et al. | BMC Infectious Diseases | 2006 |
| 3 | Update of Practice Guidelines for the Management of Community-Acquired Pneumonia in Immunocompetent Adults | Review | 876 | Mandell, L.A.,  et al. | Clinical Infectious Diseases | 2003 |
| 4 | Real-time PCR in clinical microbiology: Applications for routine laboratory testing | Review | 800 | Espy, M.J.,  et al. | Clinical Microbiology Reviews | 2006 |
| 5 | RNAi therapeutics: A potential new class of pharmaceutical drugs | Review | 785 | Bumcrot, D.,  et al. | Nature Chemical Biology | 2006 |
| 6 | Viral membrane fusion | Review | 758 | Harrison, S.C. | Nature Structural and Molecular Biology | 2008 |
| 7 | Bats: Important reservoir hosts of emerging viruses | Review | 708 | Calisher, C.H.,  et al. | Clinical Microbiology Reviews | 2006 |
| 8 | Resistance to antibiotics: Are we in the post-antibiotic era? | Review | 699 | Alanis, A.J. | Archives of Medical Research | 2005 |
| 9 | Influenza | Conference Paper | 668 | Nicholson, K.G.,  et al. | Lancet | 2003 |
| 10 | Structural bioinformatics and its impact to biomedical science | Review | 662 | Chou, K.-C. | Current Medicinal Chemistry | 2004 |
| 11 | The Severe Acute Respiratory Syndrome | Review | 538 | Peiris, J.S.M.,  et al. | New England Journal of Medicine | 2003 |
| 12 | Viral pneumonia | Review | 479 | Ruuskanen, O.,  et al. | The Lancet | 2011 |
| 13 | Are We Ready for Pandemic Influenza? | Review | 473 | Webby, R.J.,  et al. | Science | 2003 |
| 14 | Exosome function: From tumor immunology to pathogen biology | Review | 472 | Schorey, J.S.,  et al. | Traffic | 2008 |
| 15 | Perspectives on the basic reproductive ratio | Review | 472 | Heffernan, J.M.,  et al. | Journal of the Royal Society Interface | 2005 |
| 16 | Contamination, disinfection, and cross-colonization: Are hospital surfaces reservoirs for nosocomial infection? | Review | 410 | Hota, B. | Clinical Infectious Diseases | 2004 |
| 17 | Mechanisms and enzymes involved in SARS coronavirus genome expression | Review | 398 | Thiel, V.,  et al. | Journal of General Virology | 2003 |
| 18 | Severe acute respiratory syndrome | Review | 384 | Peiris, J.S.M.,  et al. | Nature Medicine | 2004 |
| 19 | Role of ventilation in airborne transmission of infectious agents in the built environment - A multidisciplinary systematic review | Review | 377 | Li, Y.,  et al. | Indoor Air | 2007 |
| 20 | Mucosal delivery of therapeutic and prophylactic molecules using lactic acid bacteria | Review | 361 | Wells, J.M.,  et al. | Nature Reviews Microbiology | 2008 |

^a^ Ranked by total citations.

^b^ The first authors showed up (on Scopus) were provided. This doesn’t mean this author contributed the most.

**Supplementary Table 2.** Top 20 countries or territories with the most documents.

| **Rank^a^** | **Country or**  **territory** | **Documents’ numbers** | **Total citations^b^** | **Citations per document^c^** | **Documents’ h-index^d^** |
| --- | --- | --- | --- | --- | --- |
| 1 | United States | 4225 | 143960 | 34.1 | 151 |
| 2 | China (mainland) | 2720 | 49316 | 18.1 | 93 |
| 3 | Hong Kong (China) | 1411 | 48828 | 34.6 | 96 |
| 4 | United Kingdom | 1134 | 39972 | 35.2 | 95 |
| 5 | Canada | 1059 | 30609 | 28.9 | 81 |
| 6 | Germany | 692 | 33698 | 48.7 | 87 |
| 7 | Taiwan (China) | 646 | 13408 | 20.8 | 50 |
| 8 | Singapore | 556 | 14523 | 26.1 | 51 |
| 9 | Australia | 488 | 14215 | 29.1 | 60 |
| 10 | Netherlands | 483 | 29629 | 61.3 | 82 |
| 11 | Saudi Arabia | 467 | 13110 | 28.1 | 53 |
| 12 | South Korea | 462 | 4969 | 10.8 | 34 |
| 13 | France | 431 | 16191 | 37.6 | 65 |
| 14 | Japan | 424 | 9478 | 22.4 | 44 |
| 15 | Italy | 316 | 10408 | 32.9 | 55 |
| 16 | Switzerland | 288 | 13714 | 47.6 | 57 |
| 17 | India | 233 | 2976 | 12.8 | 29 |
| 18 | Spain | 195 | 6019 | 30.9 | 41 |
| 19 | Egypt | 139 | 2840 | 20.4 | 24 |
| 20 | Sweden | 137 | 5603 | 40.9 | 34 |

^a^ Ranked by document number.

^b^ Total citations mean the sum of citations received these years about those documents (the *Documents’ numbers* column in this table) in each country or territory.

^c^ Citations per document was calculated according to the documents’ numbers and their total citations.

^d^ Documents’ h-index extracted from Scopus is the h-index of those documents (the *Documents’ numbers* column in this table) in the country or territory. It means there are ‘h’ documents have been cited at least ‘h’ times.

**Supplementary Table 3.** Top 20 affiliations with the most documents.

| **Rank^a^** | **Affiliation** | **Documents’ numbers** | **Total citations^b^** | **Citations per document^c^** | **Documents’ h-index^d^** |
| --- | --- | --- | --- | --- | --- |
| 1 | The University of Hong Kong | 703 | 33935 | 48.3 | 90 |
| 2 | Chinese University of Hong Kong | 499 | 14738 | 29.5 | 58 |
| 3 | Chinese Academy of Sciences | 407 | 10267 | 25.2 | 53 |
| 4 | Centers for Disease Control and Prevention | 395 | 14091 | 35.7 | 56 |
| 5 | Prince of Wales Hospital Hong Kong | 304 | 10541 | 34.7 | 50 |
| 6 | University of Toronto | 276 | 9057 | 32.8 | 42 |
| 7 | National Institutes of Health, Bethesda | 274 | 13629 | 49.7 | 70 |
| 8 | Queen Mary Hospital Hong Kong | 213 | 19399 | 91.1 | 62 |
| 9 | The University of North Carolina at Chapel Hill | 193 | 7772 | 40.3 | 54 |
| 10 | National University of Singapore | 185 | 3757 | 20.3 | 34 |
| 11 | Chinese Center for Disease Control and Prevention | 182 | 4253 | 23.4 | 35 |
| 12 | Erasmus MC | 177 | 11544 | 65.2 | 51 |
| 13 | Ministry of Health Saudi Arabia | 176 | 6345 | 36.1 | 40 |
| 14 | Institute of Microbiology Chinese Academy of Sciences | 166 | 4349 | 26.2 | 37 |
| 15 | Organisation Mondiale de la Santé | 161 | 8829 | 54.8 | 37 |
| 16 | National Institute of Allergy and Infectious Diseases | 147 | 7594 | 51.7 | 50 |
| 17 | Peking University | 136 | 2706 | 19.9 | 31 |
| 18 | Harvard Medical School | 130 | 6885 | 53.0 | 39 |
| 19 | Chinese Academy of Medical Sciences | 129 | 4052 | 31.4 | 28 |
| 20 | Fudan University | 126 | 2418 | 19.2 | 30 |

^a^ Ranked by document number.

^b^ Total citations mean the sum of citations received these years about those documents (the *Documents’ numbers* column in this table) in each affiliation.

^c^ Citations per document was calculated according to the documents’ numbers and their total citations.

^d^ Documents’ h-index extracted from Scopus is the h-index of those documents (the *Documents’ numbers* column in this table) in the affiliation. It means there are ‘h’ documents have been cited at least ‘h’ times.
